# Targeting and activation of BraATG8i by an RXLR effector DM459 contribute to downy mildew resistance in *Brassica rapa*

**DOI:** 10.1093/hr/uhaf358

**Published:** 2025-12-31

**Authors:** Ning Li, Yunyun Cao, Peirong Li, Guize Wu, Yuxin Huang, Zhijun Zhang, Xiaoyun Xin, Weihong Wang, Xiuyun Zhao, Deshuang Zhang, Yangjun Yu, Fenglan Zhang, Ning Liu, Tongbing Su, Shuancang Yu

**Affiliations:** State Key Laboratory of Vegetable Biobreeding, Beijing Vegetable Research Center (BVRC), Beijing Academy of Agriculture and Forestry Science (BAAFS), Beijing 100097, China; Key Laboratory of Biology and Genetic Improvement of Horticultural Crops (North China), Ministry of Agriculture, Beijing 100097, China; Beijing Key Laboratory of Crop Molecular Design and Intelligent Breeding, Beijing 100097, China; State Key Laboratory of Vegetable Biobreeding, Beijing Vegetable Research Center (BVRC), Beijing Academy of Agriculture and Forestry Science (BAAFS), Beijing 100097, China; Key Laboratory of Biology and Genetic Improvement of Horticultural Crops (North China), Ministry of Agriculture, Beijing 100097, China; Beijing Key Laboratory of Crop Molecular Design and Intelligent Breeding, Beijing 100097, China; College of Agriculture Science and Engineering, Shandong Agricultural University, Tai’an, Shandong 271018, China; State Key Laboratory of Vegetable Biobreeding, Beijing Vegetable Research Center (BVRC), Beijing Academy of Agriculture and Forestry Science (BAAFS), Beijing 100097, China; Key Laboratory of Biology and Genetic Improvement of Horticultural Crops (North China), Ministry of Agriculture, Beijing 100097, China; Beijing Key Laboratory of Crop Molecular Design and Intelligent Breeding, Beijing 100097, China; State Key Laboratory of Vegetable Biobreeding, Beijing Vegetable Research Center (BVRC), Beijing Academy of Agriculture and Forestry Science (BAAFS), Beijing 100097, China; Key Laboratory of Biology and Genetic Improvement of Horticultural Crops (North China), Ministry of Agriculture, Beijing 100097, China; Beijing Key Laboratory of Crop Molecular Design and Intelligent Breeding, Beijing 100097, China; State Key Laboratory of Vegetable Biobreeding, Beijing Vegetable Research Center (BVRC), Beijing Academy of Agriculture and Forestry Science (BAAFS), Beijing 100097, China; Key Laboratory of Biology and Genetic Improvement of Horticultural Crops (North China), Ministry of Agriculture, Beijing 100097, China; Beijing Key Laboratory of Crop Molecular Design and Intelligent Breeding, Beijing 100097, China; State Key Laboratory of Vegetable Biobreeding, Beijing Vegetable Research Center (BVRC), Beijing Academy of Agriculture and Forestry Science (BAAFS), Beijing 100097, China; Key Laboratory of Biology and Genetic Improvement of Horticultural Crops (North China), Ministry of Agriculture, Beijing 100097, China; Beijing Key Laboratory of Crop Molecular Design and Intelligent Breeding, Beijing 100097, China; State Key Laboratory of Vegetable Biobreeding, Beijing Vegetable Research Center (BVRC), Beijing Academy of Agriculture and Forestry Science (BAAFS), Beijing 100097, China; Key Laboratory of Biology and Genetic Improvement of Horticultural Crops (North China), Ministry of Agriculture, Beijing 100097, China; Beijing Key Laboratory of Crop Molecular Design and Intelligent Breeding, Beijing 100097, China; State Key Laboratory of Vegetable Biobreeding, Beijing Vegetable Research Center (BVRC), Beijing Academy of Agriculture and Forestry Science (BAAFS), Beijing 100097, China; Key Laboratory of Biology and Genetic Improvement of Horticultural Crops (North China), Ministry of Agriculture, Beijing 100097, China; Beijing Key Laboratory of Crop Molecular Design and Intelligent Breeding, Beijing 100097, China; State Key Laboratory of Vegetable Biobreeding, Beijing Vegetable Research Center (BVRC), Beijing Academy of Agriculture and Forestry Science (BAAFS), Beijing 100097, China; Key Laboratory of Biology and Genetic Improvement of Horticultural Crops (North China), Ministry of Agriculture, Beijing 100097, China; Beijing Key Laboratory of Crop Molecular Design and Intelligent Breeding, Beijing 100097, China; State Key Laboratory of Vegetable Biobreeding, Beijing Vegetable Research Center (BVRC), Beijing Academy of Agriculture and Forestry Science (BAAFS), Beijing 100097, China; Key Laboratory of Biology and Genetic Improvement of Horticultural Crops (North China), Ministry of Agriculture, Beijing 100097, China; Beijing Key Laboratory of Crop Molecular Design and Intelligent Breeding, Beijing 100097, China; State Key Laboratory of Vegetable Biobreeding, Beijing Vegetable Research Center (BVRC), Beijing Academy of Agriculture and Forestry Science (BAAFS), Beijing 100097, China; Key Laboratory of Biology and Genetic Improvement of Horticultural Crops (North China), Ministry of Agriculture, Beijing 100097, China; Beijing Key Laboratory of Crop Molecular Design and Intelligent Breeding, Beijing 100097, China; State Key Laboratory of Vegetable Biobreeding, Beijing Vegetable Research Center (BVRC), Beijing Academy of Agriculture and Forestry Science (BAAFS), Beijing 100097, China; Key Laboratory of Biology and Genetic Improvement of Horticultural Crops (North China), Ministry of Agriculture, Beijing 100097, China; Beijing Key Laboratory of Crop Molecular Design and Intelligent Breeding, Beijing 100097, China; State Key Laboratory of Vegetable Biobreeding, Beijing Vegetable Research Center (BVRC), Beijing Academy of Agriculture and Forestry Science (BAAFS), Beijing 100097, China; Key Laboratory of Biology and Genetic Improvement of Horticultural Crops (North China), Ministry of Agriculture, Beijing 100097, China; Beijing Key Laboratory of Crop Molecular Design and Intelligent Breeding, Beijing 100097, China; State Key Laboratory of Vegetable Biobreeding, Beijing Vegetable Research Center (BVRC), Beijing Academy of Agriculture and Forestry Science (BAAFS), Beijing 100097, China; Key Laboratory of Biology and Genetic Improvement of Horticultural Crops (North China), Ministry of Agriculture, Beijing 100097, China; Beijing Key Laboratory of Crop Molecular Design and Intelligent Breeding, Beijing 100097, China; State Key Laboratory of Vegetable Biobreeding, Beijing Vegetable Research Center (BVRC), Beijing Academy of Agriculture and Forestry Science (BAAFS), Beijing 100097, China; Key Laboratory of Biology and Genetic Improvement of Horticultural Crops (North China), Ministry of Agriculture, Beijing 100097, China; Beijing Key Laboratory of Crop Molecular Design and Intelligent Breeding, Beijing 100097, China

## Abstract

Downy mildew, caused by the biotrophic oomycete *Hyaloperonospora parasitica*, is one of the most devastating diseases affecting global *Brassica* production. Despite its significant impact, the molecular and cellular mechanisms underlying both compatible and incompatible interactions of *H. parasitica* and *Brassica rapa* remain poorly understood. In this study, we identified an *H. parasitica* RXLR effector, DM459, which demonstrates the ability to induce autophagy by targeting BraATG8i, a key component of autophagosome formation, as confirmed by multiple *in vivo* and *in vitro* assays. BraATG8i is a positive regulator of defense against downy mildew, which was determined by the *BraATG8i* overexpression and RNA interference in *B. rapa*. Furthermore, the effector DM459 interacts with BraATG8i as well as BraATG4, BraATG3, and BraATG7—core proteins required for autophagosome assembly. This interaction-enhanced autophagy contributed to elevated disease resistance. Moreover, pathogen inoculation or DM459 presence stimulated salicylic acid (SA) biosynthesis, which in turn activated *BraATG8i* expression and further elevated autophagy. Collectively, our results demonstrated that the effector DM459 triggers autophagy by directly targeting BraATG proteins and simultaneously activates SA signaling, which consequently enhances plant resistance to downy mildew.

## Introduction

Downy mildew, caused by biotrophic oomycetes, represents a major constraint to the productivity of *Brassica* crops worldwide. These pathogens primarily belong to the genera *Hyaloperonospora* and *Plasmopara*, with *Hyaloperonospora parasitica* and *H. brassicae* being the main causal agents of *Brassica* downy mildew [1]. The disease is particularly destructive under favorable environmental conditions typically encountered during spring and autumn seasons, where yield losses can exceed 50% in severe epidemics [2–4]. The management of oomycete diseases presents substantial challenges due to the remarkable ability of these pathogens to develop resistance to fungicides and evade host immunity mechanisms. This situation makes the development of resistant cultivars through breeding programs a crucial strategy for ensuring sustainable production of *Brassica* vegetables.

*Brassica rapa*, as one of the most important vegetable crops in China, has been the subject of extensive genetic studies aimed at identifying resistance sources against downy mildew. Through various genetic mapping approaches, researchers have identified a series of major quantitative trait loci associated with disease resistance [5–7]. Despite these significant advances, the molecular mechanisms governing the compatible and incompatible interactions between *H. parasitica* and *Brassica* crops remain poorly characterized. This knowledge gap significantly hinders our ability to develop effective and durable resistance strategies against this economically important pathogen.

Plants have evolved a sophisticated two-layered innate immune system to defend against pathogen attacks. The first layer, known as pathogen-associated molecular pattern (PAMP)-triggered immunity (PTI), is activated when plant pattern recognition receptors detect PAMPs. To counteract this initial defense, pathogens deliver effectors into host cells to suppress PTI. In response, plants have developed a second layer of defense called effector-triggered immunity (ETI), which is activated when resistance (R) proteins directly or indirectly recognize specific pathogen effectors [8–10]. The specific molecular interactions between pathogen effectors and plant proteins form the basis of this recognition system, making effector genes valuable tools for investigating plant immunity mechanisms [11, 12].

Oomycete pathogens employ an array of effectors to manipulate host immunity. These effectors can be classified as either apoplastic or cytoplasmic based on their site of action [13–15]. Among cytoplasmic effectors, RXLR proteins constitute the most extensively studied class, characterized by a conserved Arg–x–Leu–Arg (RXLR) motif that facilitates their entry into host cells [16, 17]. Current research indicates that while most identified host targets of RXLR effectors are negative regulators of plant immunity, some RXLR effectors conserved across diverse oomycete pathogens can be recognized by plant R proteins to activate immune responses [18–20]. Compared to the well-studied virulence-promoting effectors, characterization of the latter group and their regulatory mechanisms has received relatively less attention. However, these defense-activating effectors are of particular interest as they may serve as ideal ‘activators’ for effectively mobilizing host immunity in transgenic plants, potentially providing protection against diverse pathogens.

Recent years have witnessed significant progress in functionally characterizing RXLR effectors from different *Hyaloperonospora* species and isolates. For instance, the *Hyaloperonospora* effector HaRxLL470 enhances plant susceptibility to *H. arabidopsidis* isolate Noco2 by attenuating the DNA-binding activity of the bZIP transcription factor HY5 in *Arabidopsis* [21]. Analysis of the *grapevine* downy mildew pathogen (*Plasmopara viticola*) effector PvRXLR131, which shows over 70% sequence similarity to HaRxLL470, suggests that this conserved effector and its orthologs might function as core pathogenicity factors [22]. Interestingly, two *H. arabidopsidis* effectors, ATR13 and ATR1, can trigger RPP13- and RPP1-mediated resistance, respectively, in *Arabidopsis*, indicating their potential roles in plant defense against *H. parasitica* [23]. A recent secretome analysis of *H. parasitica* isolate BJ2020, pathogenic on cabbage (*Brassica oleracea* var. *capitata* L.), identified 276 candidate effectors, confirming their potential involvement in plant–pathogen interactions [24]. The characterized RXLR effectors in the *H. arabidopsidis* pathosystem show isolate-specific differences, with pathogens infecting *Arabidopsis* being generally nonpathogenic to other *Brassica* species. Despite these advances, no RXLR effectors in the *H. parasitica* pathosystem have been functionally characterized to date.

Autophagy, an essential intracellular degradation pathway for removing damaged or unnecessary cellular components, plays a crucial role in plant responses to biotrophic pathogens [25]. Plants utilize the autophagy pathway to regulate immunity-related programmed cell death (PCD), thereby fine-tuning the immune system [26, 27]. In *Arabidopsis*, more than 40 autophagy-related genes (ATGs) have been identified, with ATG8 family proteins representing critical players required for autophagosome biogenesis and cargo recruitment. A distinctive feature of ATG8 proteins is their tight association with autophagic membranes throughout autophagosome formation, making them reliable indicator proteins for monitoring autophagy [28]. The core mechanism involves the binding of target receptor proteins to ATG8 and the subsequent association of ATG8 with the autophagosome membrane [29–32]. Emerging evidence demonstrates that ATG8 serves as a direct target for pathogen effectors, leading to perturbation of autophagy-mediated immunity. For example, the virulence effector SDE3 from ‘*Candidatus Liberibacter asiaticus*’ inhibits autophagy in citrus, *Arabidopsis*, and tobacco, thereby negatively regulating plant resistance [33]. Similarly, the oomycete effector PexRD54 from *Phytophthora infestans* disrupts plant defense by displacing the autophagy cargo receptor Joka2 from the ATG8CL complex [34]. Collectively, these findings establish that effector-ATG8 interactions represent an important interface in plant–pathogen conflicts.

In this study, we report the functional characterization of an RXLR effector DM459 from *H. parasitica* that interacts with BraATG8i and other autophagy-related proteins both *in vivo* and *in planta*, promoting autophagosome formation. We further demonstrate that DM459 stimulates salicylic acid (SA) biosynthesis in *B. rapa*, which in turn enhances *BraATG8i* expression. Phenotypic analysis of *BraATG8i* overexpression and RNAi transgenic *B. rapa* lines provides evidence that BraATG8i functions as a positive regulator of plant immunity against *H. parasitica*. Based on our findings, we propose a model wherein DM459 interacts with BraATG8i to activate host resistance against *H. parasitica*. These results advance our understanding of ATG8-mediated immunity in plants and provide the first evidence that an oomycete RXLR effector can induce plant immune responses through the autophagy pathway.

## Results

### DM459 is a *bona fide* effector of the oomycete *H. parasitica* var *brassicae*

Genome sequencing of the *H. parasitica* isolate used in this study revealed 51 putative RXLR effectors. Among them, the expression of effector *DM459* was strongly induced in resistant plants (CC031^R^), compared to the susceptible plants (CC032^S^), when plants were challenged with *H. parasitica*, indicating that DM459 might play an important role in *H. parasitica* infections (Fig. S1B). The basic physicochemical information of DM459 is listed in Table S2. DM459 encodes a putative protein of 362 amino acids, with a predicted signal peptide at the N-terminus followed by the RXLR motif. AlphaFold2 predicts the 3D structure of its functional domains: an N-terminal secretory signal peptide (residues 1–24), a central disordered region likely mediating host interactions, and a C-terminal effector domain containing the conserved RXLR motif (Table S3 and Fig. S1A). A phylogenetic tree was constructed on the basis of protein sequences of DM459 and other selected RXLR effectors. DM459 was robustly grouped in a distinct subclade with *Arabidopsis thaliana* Recognized 13 (ATR13) and other related effectors, providing phylogenetic evidence that DM459 belongs to the RXLR effector family (Fig. S1C).

**Figure 1:**
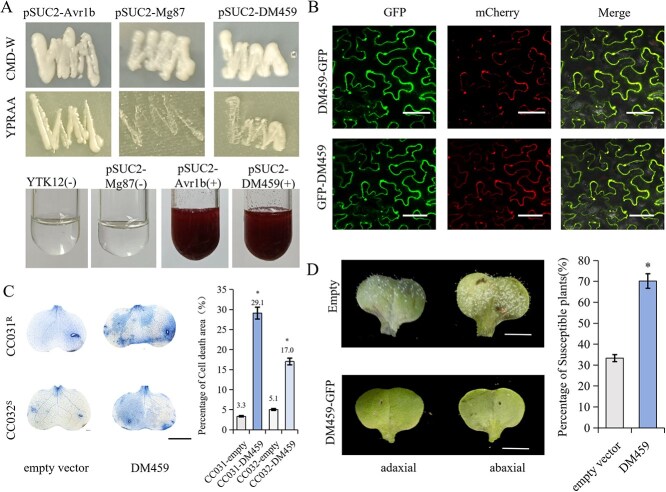
DM459 functions as an effector protein that induces cell death in *B. rapa.* (A) Secretion of DM459 was confirmed in the yeast signal sequence trap system (strain YTK12). Yeast cells expressing the pSUC2-DM459 fusion grew on YPRAA medium and turned red in the presence of TTC, comparable to the positive control (pSUC2-Avr1b). Negative controls (YTK12, empty pSUC2 vector, and pSUC2-Mg87) failed to induce a color change. (B) Subcellular localization of DM459 in *N. benthamiana*. GFP-DM459 (green) or DM459-GFP was co-expressed with mCherry-tagged markers for the nucleus and plasma membrane (red). Images were acquired 48–72 hours post-agroinfiltration. Bars, 100 μm. (C) DM459 induces cell death in *B. rapa* cotyledons. Cell death was detected by trypan blue staining 48 hours after transient expression of the *35S-DM459-GFP* construct. Bars, 0.5 cm (*n* = 20). (D) Transient expression of *DM459* enhances resistance to *H. parasitica* in *B. rapa*. Cotyledons expressing DM459-GFP or an empty vector (control) were inoculated with *H. parasitica*. Disease symptoms were recorded at 7 dpi. Bars, 0.5 cm (*n* = 30).

Oomycetes can secrete RXLR effectors that manipulate immune responses outside or inside host cells [35, 36]. To confirm whether DM459 is a secreted protein, the predicted signal peptide of DM459 was examined using a genetic assay based on the requirement of yeast cells for invertase secretion to grow on a raffinose medium [37]. The predicted signal peptide sequence of DM459 was fused to the pSUC2 vector, and the constructs pSUC2-DM459, positive control pSUC2-Avr1b*,* and negative control pSUC2-Mg87 were introduced to the YTK12 yeast strains separately. Both yeast strains harboring pSUC2-DM459 and pSUC2-Avr1b constructs enabled the YTK12 yeast to grow on CMD-W medium and YPRAA medium, and concurrently, 2,3,5-triphenyltetrazolium chloride (TTC)-treated cultures of those yeast strains exhibited red colors, indicating pSUC2-DM459 possesses invertase enzymatic activity. These results strongly suggest that DM459 could be secreted from *H. parasitica*. On the contrary, yeast strains harboring pSUC2-Mg87 could not survive in the YPRAA medium, and their cultures remained colorless in the presence of TTC (Fig. 1A). Thus, our results collectively demonstrate that DM459 is a secretory protein, consistent with the characteristics of known RXLR effectors.

To determine the subcellular localization of DM459, an N-terminal green fluorescent protein (GFP) fusion to DM459 was transiently expressed in *Nicotiana benthamiana*. According to the distribution and intensity of GFP signals, both DM459-GFP and GFP-DM459 fusion proteins accumulated strongly in the plasma membrane and nucleoplasm, with additional expression in the cytoplasm (Fig. 1B). Notably, DM459 exhibited a distinct punctate pattern within the cytoplasm, a localization characteristic that may be relevant to its functional role upon internalization (Fig. S1D). Western blot analysis further confirmed that both the DM459-GFP fusion protein and the GFP control were expressed as full-length proteins in tobacco cells, with no signs of proteolytic degradation (Fig. S1E).

To evaluate the function of DM459 during infection, DM459 was transiently expressed in the cotyledon leaves of CC032^S^ and CC031^R^ lines by the agroinfiltration method. Trypan blue staining suggested that DM459 could induce significant cell death in the DM459 cotyledon leaves after 2 days of treatment; however, cell death was hardly observed in the control leaves in either CC032^S^ or CC031^R^ lines (Fig. 1C). The results suggested that DM459 could trigger the hypersensitive response (HR) in the leaves.

To further investigate the role of DM459 in *H. parasitica* virulence, the *35S-DM459-GFP* plants were also sprayed with spore solutions of *H. parasitica*. After 1 week of treatment, the mildew layer was significantly decreased or almost absent in the cotyledon leaves with DM459 transiently expressed. In contrast, downy grayish mold on the abaxial surface of leaves could be observed in the control group (Figs 1D and S2D). Nearly 80% of the *35S-DM459-GFP* seedlings were resistant to *H. parasitica,* whereas the control seedlings were 100% susceptible, indicating that DM459 could enhance plant resistance to *H. parasitica*. Together, these results suggested that *DM459* might encode a cytoplasmic RXLR effector that plays a positive role in *B. rapa* resistance to *H. parasitica*.

### Effector DM459 interacts with autophagy protein BraATG8i

**Figure 2:**
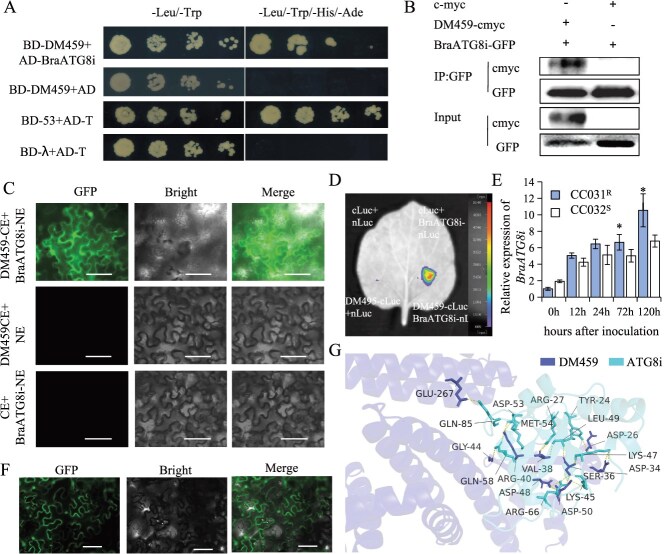
The effector DM459 physically interacts with BraATG8i both *in vivo* and *in vitro.* (A) Y2H assay. Physical interactions between pGBKT7-DM459 (BD-DM459) and pGADT7-BraATG8i (AD-BraATG8i) were examined on SD dropout medium. (B) Co-IP assay. Proteins were extracted from *N. benthamiana* leaves co-expressing DM459-cmyc and BraATG8i-GFP. Immunoprecipitation was performed using an anti-GFP antibody, followed by immunoblotting with anti-cmyc and anti-GFP antibodies. (C) BiFC assay. DM459-CE and BraATG8i-NE were co-expressed in *N. benthamiana* leaves. Reconstituted GFP fluorescence was observed by confocal microscopy 3 days after infiltration. Bars, 100 μm. (D) LCI assay. DM459-cLuc and BraATG8i-nLuc were co-infiltrated into *N. benthamiana* leaves. Luciferase activity was detected using a CCD imaging system. (E) Expression profiles of *BraATG8i* in response to *H. parasitica* infection. Transcript levels of *BraATG8i* were quantified by qRT-PCR in the resistant (CC031^R^) and susceptible (CC032^S^) *B. rapa* lines at the indicated hours postinoculation (hpi). Data are presented as mean ± SD (*n* = 5 biological replicates with three technical replicates each). Different letters indicate significant differences (*P* < 0.05, Duncan’s test). *GAPDH* was used as an internal control. (F) Subcellular localization of BraATG8i. BraATG8i-GFP was transiently expressed in *N. benthamiana* leaves. Fluorescence was observed by confocal microscopy 3 days postinfiltration. Bars, 100 μm. (G) Structural prediction of the DM459–BraATG8i interaction by AlphaFold3.

To identify potential host targets of DM459, a yeast two-hybrid (Y2H) prey library was constructed using RNA extracted from CC032^S^ leaves at 0, 12, 72, and 120 hours postinfection with *H. parasitica*. The ‘prey’ library was co-transformed with a GAL4 DNA-binding domain-DM459 fusion ‘bait’ construct, leading to the identification of several DM459-interacting candidates (Table S4). Subsequently, ‘One-to-One’ Y2H validation confirmed that BraA05g031480.3C might be the primary candidate that interacted with the effector DM459 (Fig. 2A). BraA05g031480.3C encodes a putative 115-amino acid protein exhibiting high sequence similarity to *Arabidopsis thaliana* Autophagy-related 8 (AtATG8) proteins. Phylogenetic analysis indicated that BraA05g031480.3C is the ortholog of AtATG8i, and it was accordingly designated BraATG8i (Fig. S2A).

**Figure 3:**
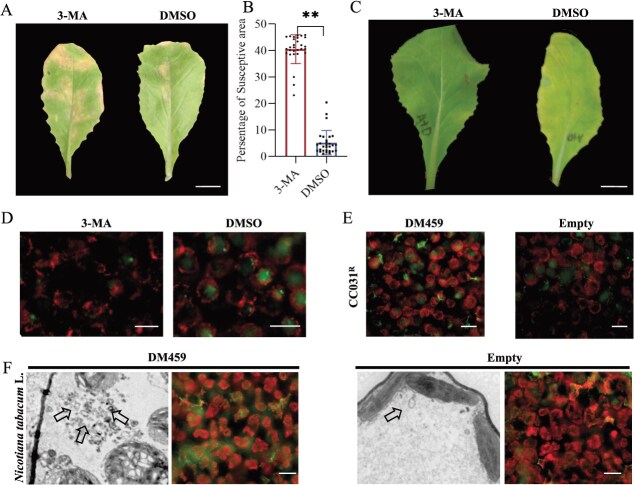
Autophagy contributes to disease resistance in *B. rapa* and is modulated by DM459. (A) Autophagy inhibition increases susceptibility to downy mildew. Leaves of the susceptible line CC032^S^ were treated with the autophagy inhibitor 3-MA or a DMSO control, followed by inoculation with *H. parasitica*. Representative leaves from each treatment are shown. Bar, 1 cm. (B) Quantification of disease susceptibility. The percentage of diseased leaf area was measured and calculated from 30 leaves per treatment. Data are presented as mean ± SD (*n* = 30). Statistical significance was determined by a two-tailed Student’s *t*-test (***P < 0.001). (C) Autophagy inhibition suppresses DM459- and BraATG8i-induced cell death. Cell death induced by co-expression of DM459 and BraATG8i in *B. rapa* cotyledons was suppressed by pretreatment with 3-MA (*n* = 20). Bar, 1 cm. (D) 3-MA treatment suppresses basal autophagy in *B. rapa*. Autophagic structures in *B. rapa* leaf cells treated with 3-MA or DMSO were visualized by MDC staining and observed via confocal microscopy. Bar, 50 μm. (E) Transient expression of *DM459-cmyc* induces autophagy in *B. rapa*. The resistant line CC031^R^ was transiently transformed with DM459-cmyc or a cmyc control. Autophagic flux was monitored by MDC staining. Bar, 50 μm. (F) DM459 expression induces autophagy in *N. benthamiana*. Autophagosome formation in *N. benthamiana* leaves expressing DM459-GFP was confirmed by both TEM and MDC staining. Bar, 50 μm.

The physical interaction between DM459 and BraATG8i was further validated by co-immunoprecipitation (Co-IP), luciferase complementation imaging (LCI), and bimolecular fluorescence complementation (BiFC) experiments. In Co-IP experiments, transient co-expression of BraATG8i-GFP and DM459-cmyc in *N. benthamiana* leaves followed by immunoprecipitation with anti-GFP agarose gels demonstrated specific pull-down of DM459-cmyc, but not the cmyc control, with BraATG8i-GFP, where all constructs were detected in the input fractions (Fig. 2B). The BiFC analysis revealed strong GFP fluorescence exclusively in leaves co-expressing DM459-CE and BraATG8i-NE fusion proteins, with no signal observed in negative controls (Fig. 2C). Similarly, LCI assays detected luminescence signals only in cells co-expressing DM459-cLuc and BraATG8i-nLuc, further supporting a direct interaction between DM459 and BraATG8i (Fig. 2D). AlphaFold3 prediction of the DM459–BraATG8i complex structure showed localized interaction interfaces, which were abolished by mutating BraATG8i (Figs 2G and S2E), thereby confirming the specificity of this interaction. Together, these *in vitro* and *in vivo* results are complemented by computational evidence, firmly establishing the DM459–BraATG8i interaction.

The quantitative polymerase chain reaction (qPCR) analysis demonstrated a progressive increase in *BraATG8i* expression up to 5 days postinoculation (dpi) with *H. parasitica* (Fig. 2E). Notably, transcript accumulation was significantly higher in the resistant line CC031^R^ compared to the susceptible line CC032^S^, suggesting the potential association between *BraATG8i* expression and pathogen resistance. Subcellular localization studies revealed that BraATG8i-GFP localized to the nucleus and cytoplasmic membrane (Fig. 2F), consistent with previous reports on other ATG8 proteins. To exclude potential artifacts from C-terminal cleavage, we confirmed that GFP-BraATG8i exhibited identical localization patterns (Fig. 2F). These data collectively indicate that *BraATG8i,* whose expression is strongly induced upon pathogen challenge, may contribute to *B. rapa* resistance against *H. parasitica*.

Given the presence of 17 *BraATG8* genes in the *B. rapa* genome, we next investigated whether DM459 could interact with additional BraATG8 family members. Transcriptome data indicated that 12 *BraATG8* genes were expressed in leaf tissues (Fig. S2B). Among them, most *BraATG8s*, including *BraATG8i,* exhibited significant upregulation upon *H. parasitica* infection, implicating their potential role in plant defense. Three highly expressed *BraATG8* genes in CC031^R^-*BraA03g023610.3C* (*BraATG8g*), *BraA03g042050.3C* (*BraATG8d*), and *BraA03g049890.3C* (*BraATG8a*) were randomly selected for BiFC analysis (Fig. S2B). GFP fluorescence was observed in *N. benthamiana* cells co-expressing DM459 with each of these BraATG8 proteins, and their subcellular localization patterns mirrored those of the DM459–BraATG8i interaction (Fig. S2C). These results suggest that DM459 may exhibit broader interaction specificity with multiple BraATG8 family members in host cells.

### DM459 induces autophagy and improves the resistance of *Brassica rapa* to downy mildew

To investigate the potential role of autophagy in plant defense against *H. parasitica*, *B. rapa* leaves were pretreated with the autophagy inhibitor 3-methyladenine (3-MA), which suppresses autophagosome formation, prior to pathogen inoculation. At 5 dpi, 3-MA-treated leaves showed significantly enhanced susceptibility to *H. parasitica*, as indicated by larger lesion areas compared to untreated controls (Fig. 3A and B). To further assess whether autophagy contributes to DM459–BraATG8i-mediated resistance, we evaluated the effect of 3-MA under co-expression conditions. Consistent with our hypothesis, 3-MA treatment compromised disease resistance even when *DM459* and *BraATG8i* were co-expressed (Fig. 3C).

**Figure 4:**
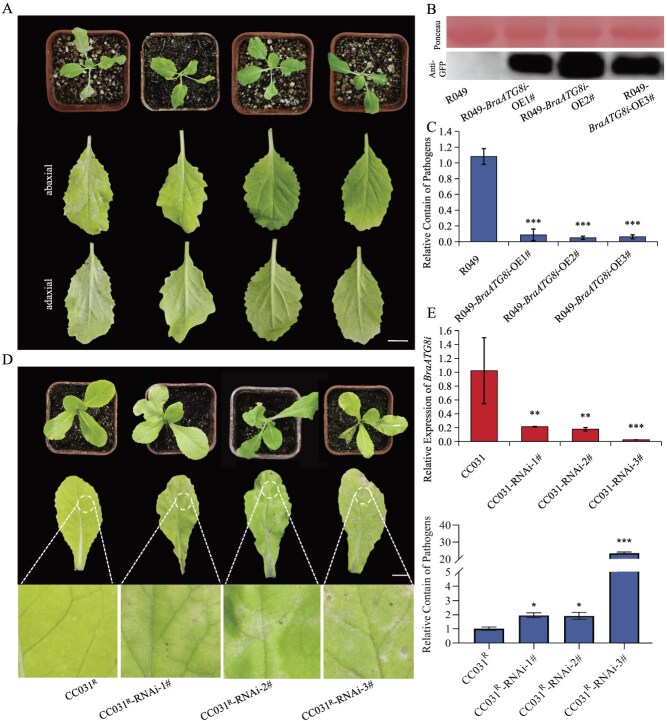
*BraATG8i* enhances *B. rapa* resistance to downy mildew. (A) Overexpression of BraATG8i enhances disease resistance. T_1_ generation *BraATG8i*-overexpressing plants in the susceptible background R049 and corresponding wild-type controls were inoculated with *H. parasitica* at the two- to three-leaf stage. Disease symptoms were photographed and assessed at 7 dpi. Bar, 1 cm. (B) Immunoblot confirmation of BraATG8i overexpression. Protein extracts from T_1_ overexpression lines were analyzed by western blot using an anti-GFP antibody to detect the BraATG8i-GFP fusion protein. (C) Pathogen biomass quantification in *BraATG8i* knockdown lines. Fungal biomass in T_1_  *BraATG8i*-overexpressing lines (in susceptible background CC032^S^) and controls was measured by qRT-PCR analysis of the *H. parasitica* EST (ethylene-forming enzyme) transcript level at 7 dpi. (D) *BraATG8i* knockdown enhances disease susceptibility. T_1_  *BraATG8i*-RNAi plants and controls were inoculated with *H. parasitica*. Representative leaf phenotypes were recorded at 7 dpi. Bar, 1 cm. (E) *BraATG8i* expression level and pathogen biomass quantification in transgenic plants. The transcript levels of *BraATG8i* in the T_1_ RNAi lines were validated by qRT-PCR. *GAPDH* (*BraA06g009730.3C*) was used as an internal reference gene. Data are presented as mean ± SD from three biological replicates. Fungal biomass in T_1_ BraATG8i-RNAi lines (in resistant background CC031^R^) and controls was measured as (C).

We next examined autophagy activity during infection using monodansylcadaverine (MDC) staining to visualize autophagic structures. *H. parasitica*-inoculated leaves exhibited a pronounced increase in MDC-labeled puncta relative to 3-MA-treated samples (Fig. 3D). Together, these data imply that DM459 may interfere with BraATG8i-dependent autophagy to subvert plant immunity.

To determine whether DM459 directly activates autophagy, we transiently expressed DM459 in *B. rapa* leaves to observe its influence on autophagosome formation. MDC staining showed a marked increase in fluorescent puncta in *DM459*-expressing leaves, indicative of enhanced autophagic activity (Figs 3E and S3A). Similarly, when *DM459* was expressed in *N. benthamiana*, transmission electron microscopy (TEM) revealed a higher number of autophagic bodies, and MDC staining further confirmed elevated autophagic flux (Fig. 3F). Comparative analysis of MDC-stained autophagic structures in resistant and susceptible genotypes following *H. parasitica* infection revealed distinct morphological patterns (Fig. S3B). Collectively, these results support a model in which DM459-triggered autophagy plays a critical role in modulating plant resistance to *H. parasitica*.

### BraATG8i is a positive regulator of plant immunity

Our previous study showed that *BraATG8i* expression was strongly induced following *H. parasitica* infection (Fig. 2E). To explore its functional relevance during pathogen challenge, we first performed transient expression assays in two *B. rapa* lines. Transient expression of *BraATG8i-GFP* in *B. rapa* cotyledons led to enhanced resistance to *H. parasitica* compared with GFP-only controls (Fig. S4), indicating a potential role for BraATG8i in pathogen defense.

To further assess the defensive function of BraATG8i, we generated stable transgenic *B. rapa* plants overexpressing *BraATG8i* (*BraATG8i*-OE). Due to the low genetic transformability of line CC032^S^, we used the susceptible cultivar R049 for transgenic experiments. Three independent *BraATG8i*-OE lines were generated, and randomly selected T_1_ lines were used in pathogen challenge assays. Western blot analysis confirmed the accumulation of BraATG8i-GFP fusion proteins in these lines (Fig. 4B), with no obvious morphological alterations observed (Fig. 4A). Pathogenicity assays revealed that *BraATG8i*-OE plants displayed significantly enhanced resistance to *H. parasitica*, as indicated by reduced pathogen growth and smaller lesion areas compared to wild-type R049 plants (Fig. 4A and C). These results demonstrate that *BraATG8i* overexpression enhances resistance to *H. parasitica* infection.

To complement these gain-of-function studies, we attempted to knock out *BraATG8i* in the resistant line CC031^R^ using CRISPR-Cas9. However, the knockout lines showed resistance levels similar to those of CC031^R^, possibly due to functional redundancy among BraATG8 family members. We therefore adopted an RNA interference (RNAi) approach and successfully generated three independent *BraATG8i*-RNAi transgenic lines, in which *BraATG8i* expression was specifically downregulated, as verified by quantitative reverse transcription PCR (qRT-PCR) (Fig. 4E). Pathogen infection assays conducted with these lines yielded phenotypes opposite to those of *BraATG8i*-OE plants: *BraATG8i*-silenced plants developed significantly larger lesions and supported increased pathogen growth compared to wild-type CC031^R^ plants (Fig. 4D and E). Taken together, our genetic evidence from both overexpression and RNAi-based silencing strongly supports a positive regulatory role for BraATG8i in *B. rapa* immunity against *H. parasitica*.

### DM459 also interacts with BraATG4, BraATG3 and BraATG7

**Figure 5:**
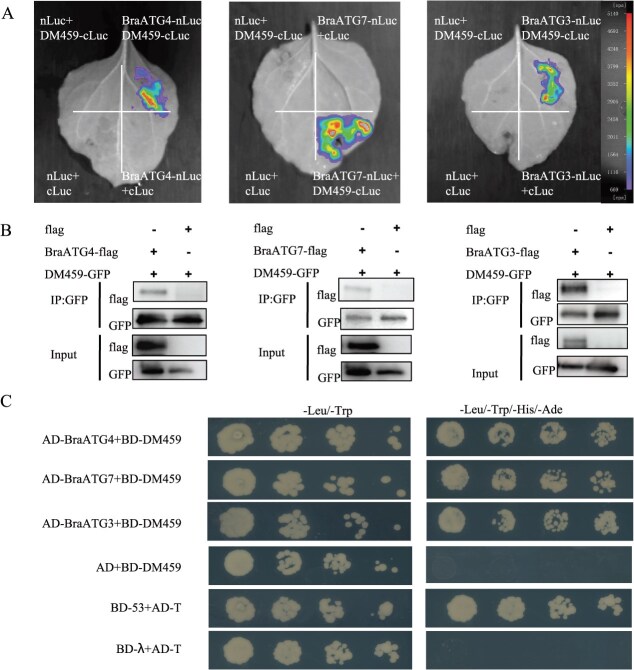
The effector DM459 physically interacts with multiple BraATG proteins in *B. rapa*. (A) LCI assay. Interactions between DM459 and BraATG4, BraATG7, or BraATG3 were tested *in planta* by co-expressing the indicated protein pairs fused to complementary luciferase fragments in *N. benthamiana* leaves. (B) Co-IP assay. Protein extracts from *N. benthamiana* leaves co-expressing DM459-GFP with BraATG4-flag, BraATG7-flag, or BraATG3-flag were immunoprecipitated (IP) with an anti-GFP antibody, followed by immunoblotting (IB) with the indicated antibodies. (C) Y2H assay. Direct interactions were tested in yeast. DM459 was fused to the DNA-binding domain (BD), while the BraATG proteins were fused to the activation domain (AD). Protein–protein interactions enable growth on selective medium (−LWH) and activate reporter genes.

During autophagosome biogenesis, ATG8 family proteins sequentially interact with ATG4, ATG7, and ATG3 to form distinct complexes, ultimately anchoring to autophagosomal membranes to facilitate membrane expansion [29, 32]. Since DM459 directly binds BraATG8i and induces autophagy (Fig. 2), we asked whether DM459 also targets other core autophagy components. LCI assays revealed strong luminescence when DM459-cLuc was co-expressed with BraATG4-nLuc, BraATG3-nLuc, or BraATG7-nLuc in *N. benthamiana* leaves, whereas control combinations with empty vectors produced no signal (Fig. 5A), indicating that DM459 physically associates with multiple BraATG8-interacting proteins. To further verify these interactions, Co-IP was performed. DM459-GFP and N-terminal flag-tagged versions of BraATG4, BraATG3, or BraATG7 were co-expressed in *N. benthamiana*, with a flag-tagged empty vector as a negative control. Anti-GFP pull-down specifically recovered each BraATG-flag protein when co-expressed with DM459-GFP, but not in controls (Fig. 5B). All fusion proteins were detected in input samples, confirming their expression. Y2H assays were conducted for additional confirmation. Positive interactions were indicated by yeast growth on selective medium lacking Leu/Trp/His/Ade (Fig. 5C). Additionally, AlphaFold3 independently predicted the molecular interactions of DM459 with these proteins (Fig. S5). Together, these results establish that DM459 directly interacts not only with BraATG8i but also with multiple autophagy core components, including BraATG4, BraATG7, and BraATG3.

### DM459 significantly potentiated the interaction between BraATG8i and the selected BraATGs

**Figure 6:**
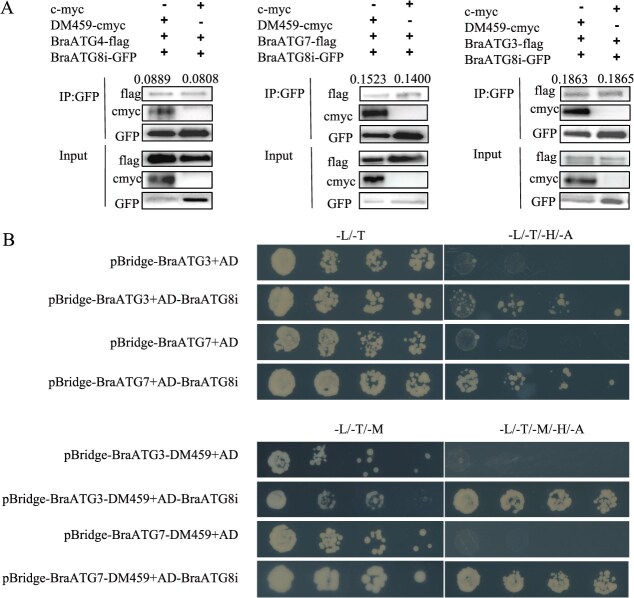
The effector DM459 enhances autophagosome formation by strengthening interactions within the core autophagy machinery. (A) DM459 potentiates the association between BraATG8i and core ATG components *in planta*. Co-IP assays were performed on protein extracts from *N. benthamiana* leaves co-expressing BraATG8i-GFP with BraATG4-Flag, BraATG7-Flag, or BraATG3-Flag, in the presence of DM459-cmyc or a cmyc control. The presence of DM459 strengthened the interactions between BraATG8i and the indicated BraATG proteins. Relative band intensity from the co-precipitated fractions, quantified from three independent replicates, is indicated above the blots. The numbers show the relative interaction intensity. (B) Y3H assay confirms DM459-mediated enhancement of protein interactions. Direct enhancement of the BraATG8i-BraATG3 and BraATG8i-BraATG7 interactions by DM459 was confirmed in yeast. The third plasmid expressing DM459 (or an empty vector control) was introduced into yeast cells expressing the BraATG8i and BraATG3/7 pairs. Protein interaction strength was assessed by growth on selective medium and reporter gene activation.

Previous studies confirmed that DM459 directly interacts with BraATG8i and other BraATG8-binding proteins, and that its overexpression or *H. parasitica* infection induces host autophagy. Based on these findings, we hypothesized that DM459 enhances autophagic activity by binding directly to BraATG proteins. To test this hypothesis, the Co-IP assays were conducted to explore whether DM459 could influence the interactions between BraATG8i and other BraATG8-interacting proteins, such as BraATG4, BraATG7, and BraATG3. Consistent with prior reports, we confirmed that BraATG8i binds directly to each of these proteins. Notably, the presence of DM459 substantially strengthened the binding between BraATG8i and BraATG4 or BraATG7, whereas its effect on the BraATG8i–BraATG3 interaction was only marginal (Fig. 6A).

To further validate DM459’s role in promoting BraATG8i complex formation, we used a yeast three-hybrid (Y3H) assay. Yeast cells co-expressing pBridge-BraATG3/BraATG7-DM459 and pGADT7-BraATG8i showed enhanced growth on selective medium (−Leu/−Trp/−Met/−His/−Ade and − Leu/−Trp/−His/−Ade) compared to controls expressing pBridge-BraATG3/BraATG7 without DM459 (Fig. 6B), indicating that DM459 facilitates molecular interactions between BraATG8i and its partners, particularly BraATG7. Together, these results support a model in which DM459 promotes BraATG8i–BraATG7 complex assembly, thereby facilitating autophagosome formation during *H. parasitica* infection.

### DM459 activates autophagy partially through SA signaling

**Figure 7:**
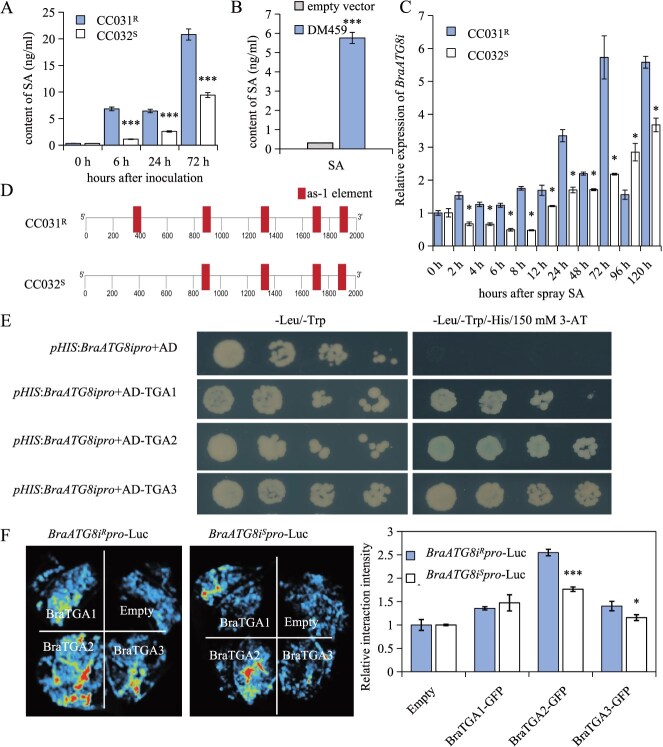
SA signaling contributes to *BraATG8i*-mediated resistance against downy mildew. (A) Endogenous SA accumulation in response to *H. parasitica* infection. Endogenous SA levels in the susceptible (CC032^S^) and resistant (CC031^R^) *B. rapa* lines were quantified by liquid chromatography–mass spectrometry (LC–MS) at the indicated hours postinoculation with *H. parasitica*. Data are presented as mean ± SD (*n* = 5 biological replicates). (B) The effector DM459 induces SA biosynthesis. Endogenous SA content was measured by LC–MS in *B. rapa* cotyledons 3 days after transient expression of DM459-GFP or a GFP control. Data are presented as mean ± SD (*n* = 5 biological replicates). (C) SA treatment induces *BraATG8i* expression. *BraATG8i* transcript levels in CC032^S^ and CC031^R^ lines were analyzed by qRT-PCR at various time points after foliar application of 20 mM SA. *GAPDH* was used as an internal control. Data are presented as mean ± SD (*n* = 5 biological replicates). (D) Schematic representation of the *as-1* cis-element in the *BraATG8i* promoter. Diagram shows the location of the predicted *as-1* element in the promoter regions of both CC031^R^ and CC032^S^ lines. (E) BraTGA transcription factors bind the *BraATG8i* promoter *in vitro*. Y1H assay demonstrates the binding of BraTGA proteins to the promoter sequence of *BraATG8i*. Growth on selective medium (−LWH + Aureobasidin A) indicates a positive interaction. (F) BraTGA transcription factors bind the *BraATG8i* promoter *in planta*. LCI assay confirms the interaction between BraTGA proteins and the *BraATG8i* promoter in *N. benthamiana* leaves.

SA is a key phytohormone regulating plant defense against pathogen invasion [38–40]. To assess its role in *H. parasitica* infection, we quantified SA levels in susceptible (CC032^S^) and resistant (CC031^R^) *B. rapa* lines. Both genotypes showed increased SA accumulation after infection, though CC032^S^ exhibited significantly lower levels than CC031^R^ (Fig. 7A), indicating genotype-specific differences in SA biosynthesis. Transient expression of DM459 in cotyledons also induced SA accumulation, albeit to a lesser degree than pathogen infection (Fig. 7B), suggesting that DM459 partially activates SA-dependent defense. Correspondingly, qRT-PCR analysis revealed reduced expression of SA biosynthesis (*ICS1*, *EDS5*) and signaling-related genes (*SARD*, *NPR*) in CC032^S^ compared to CC031^R^ (Fig. S6A), supporting the notion that impaired SA perception and biosynthesis contribute to the contrasting resistance phenotypes.

Promoter sequence analysis of *BraATG8i* further identified an additional *as-1* cis-element at −1601 bp exclusively in CC031^R^ (Fig. 7D), while GUS staining confirmed the promoter’s functionality in *B. rapa* (Fig. S6B). TGA transcription factors, which bind the *as-1* motif, are central regulators of SA signaling and autophagy. RNA-seq data showed that multiple *BraTGA* genes (*BraTGA1*, *BraTGA2*, *BraTGA3*) were significantly upregulated upon *H. parasitica* infection (Fig. S6A and C). Yeast one-hybrid (Y1H) assays using the *BraATG8i* promoter from CC032^S^ demonstrated specific binding of BraTGA proteins, as indicated by growth on histidine-deficient medium (Fig. 7E). Luciferase assays further revealed that co-expression of *35S-BraTGA* with either *BraATG8i^S^pro*-Luc or *BraATG8i^R^pro*-Luc enhanced luminescence, with significantly stronger signals from the resistant-type promoter (Fig. 7F). Together with the SA-induced expression of *BraATG8i* (Fig. 7C), these findings support a model in which enhanced SA accumulation and the presence of an additional *as-1* element in the *BraATG8i* promoter synergistically contribute to BraATG8i induction during *H. parasitica* infection.

## Discussion

### DM459 encodes a novel *H. parasitica* effector

RXLR effectors constitute a class of secreted proteins that play essential roles in subverting host immunity and are widely distributed across oomycete pathogens, including species of *Phytophthora*, *Hyaloperonospora*, and *Plasmopara*. These effectors display diverse subcellular localization patterns, with some residing in the apoplast and others entering plant cells to target specific organelles [13–15]. Although their classification remains debated, most RXLR effectors share a conserved architecture: an N-terminal signal peptide, a central RXLR motif often followed by an acidic region, and a C-terminal effector domain responsible for host target recognition. Research on the identification and functional characterization of RXLR effectors has advanced considerably over the past two decades.

Genomic surveys using hidden Markov models and sequence-based screening have identified large RXLR repertoires in several oomycetes, including 563 in *P. infestans* and 134 in *H. arabidopsidis*. A recent secretome study of the *H. parasitica* isolate BJ2020, which infects *B. oleracea* var. *capitata* L., predicted 65 RXLR candidates [24]. In our work, genome sequencing of a Beijing isolate pathogenic to *B. rapa* uncovered 51 RXLR-like genes upregulated during infection (unpublished data). Among these, DM459 showed consistently high expression in both susceptible and resistant *B. rapa* lines after inoculation (Fig. S1B), pointing to its potential importance in host–pathogen interplay. Phylogenetic analysis further placed DM459 in a clade with known avirulence effectors (Fig. S1C), suggesting it could trigger immune responses. Structural modeling via AlphaFold2 revealed that DM459 contains several solvent-exposed interfaces, including hydrophobic patches and charged grooves (Fig. S1A and Table S3). These features, typical of promiscuous binding proteins, may enable DM459 to interact with multiple host targets, which might facilitate the assembling of the immune-activating complexes and consequently contribute to plant resistance. The integration of evolutionary and structural data thus offers a plausible mechanism by which DM459 modulates plant immunity.

The yeast invertase assays confirmed that DM459 contains a functional secretion signal, suggesting DM459 could be secreted by *H. parasitica*. Transient expression of *DM459* in *B. rapa* cotyledons induced PCD, which indicates that DM459 might be recognized by plant immune receptor(s) and could trigger HR in plants. Subcellular localization assays showed that DM459-GFP was targeted to the nucleus, plasma membrane, and cytoplasm, and importantly, puncta formation could be observed in the cytoplasm (Fig. 1B), which implies its roles in engaging host targets across multiple compartments. Interestingly, although DM459 lacks clear homologs in *P. infestans* or *Plasmopara* genomes, it shares high similarity in RXLR-flanking regions with two *H. arabidopsidis* effectors, ATR1 and ATR13, indicating evolutionary conservation within *Hyaloperonospora*. Indeed, like ATR13, DM459 could enhance host resistance, implying it might be an avirulence effector. Together, these results suggest DM459 as a novel RXLR effector that promotes plant resistance in *B. rapa*.

Although DM459 enhances host resistance, it is still retained in the *H. parasitica* genome, presumably due to its potential roles in regulating pathogen growth; a similar observation was reported in the ATR13 effector. Additionally, it is plausible that other NLR proteins, unidentified yet in *B. rapa*, could recognize DM459 and contribute to ETI activation. Thus, future work will focus on identifying NLRs that directly interact with DM459, which might provide insight into the molecular mechanisms of biotrophic pathogenesis and host defense.

### DM459 activates autophagy by interacting with BraATG8i and other selected BraATGs, reflecting the coevolution between the host plant and the pathogen

Autophagy is an evolutionarily conserved recycling mechanism that degrades cytoplasmic components and damaged organelles to maintain cellular homeostasis [27]. Growing evidence indicates that microbial pathogens actively manipulate this process through effectors to subvert plant immunity. Bacterial pathogens exemplify diverse autophagy modulation strategies: *Pseudomonas syringae* pv. *tomato* employs HrpZ1 oligomerization to promote autophagy via ATG4b-mediated ATG8 processing, while simultaneously deploying HopF3 and AvrPtoB to suppress host autophagy through ATG8 binding and ATG1 kinase inhibition, respectively [41]. Similarly, *Candidatus Liberibacter asiaticus* effector SDE3 promotes CsATG8 degradation to inhibit autophagy [33]. These findings illustrate that autophagy can exert both antipathogen and propathogen functions in host immunity.

In fungal-plant interactions, oomycete RXLR effectors exhibit distinct autophagy subversion mechanisms. *Phytophthora infestans* effector PexRD54 competitively binds ATG8CL, displacing the positive regulator Joka2 to suppress autophagosome formation and promote infection [34]. Notably, current literature suggests that fungal RXLR effectors predominantly hijack host autophagy to enhance virulence, with no reported instances of these effectors conferring antipathogen effects. This dichotomy between bacterial and fungal effector strategies underscores fundamental differences in microbial pathogenesis.

Our study demonstrates a direct physical interaction between the oomycete effector DM459 and the autophagy-related protein BraATG8i, supported by multiple *in vivo* and *in vitro* assays. Subcellular localization revealed the co-localization of BraATG8i-GFP and DM459-GFP in both cytoplasmic and nuclear compartments, suggesting that DM459 might be involved in the autophagic activity in the infected cells. Using MDC staining to monitor autophagic flux, we observed significant induction of autophagy in *H. parasitica*-infected *B. rapa* leaves, which could be effectively suppressed by the autophagy inhibitor 3-MA. Therefore, these findings support a model wherein DM459–BraATG8i interaction modulates host autophagy to enhance resistance.

Molecular characterization revealed that DM459 interacts not only with BraATG8i but also with multiple core autophagy components, including BraATG3, BraATG4, and BraATG7. These interactions were further analyzed through structural predictions via AlphaFold3, which indicated that the binding interfaces primarily localized to the region of DM459 signal peptide (Fig. S5). This finding implies a potential noncanonical role for the effector’s signal peptide, possibly contributing to autophagy induction. Consistent with this molecular interplay, the observed co-localization of DM459 and autophagosome strongly suggests that these vesicular structures might represent functional sites for DM459 activity. It is possible that DM459 might stabilize protein complexes involved in the lipidation and membrane anchoring of ATG8, a cornerstone of autophagosome formation. Hence, our results provide mechanistic insight into how DM459 may orchestrate the assembly of ATG proteins during autophagosome biogenesis.

Our protein–protein interaction analyses suggest that DM459, BraATG8i, and associated BraATGs may assemble into a functional ternary complex. Importantly, DM459 enhances the binding affinity between BraATG8i and its interacting proteins, facilitating the autophagosome formation and subsequent host resistance. Co-IP experiments indicate that the DM459–BraATG8i complex promotes autophagosome assembly by strengthening BraATG8i interactions with BraATG4 and BraATG7 (Fig. 6). Although the dynamic nature of these complexes precluded quantitative affinity measurements *in vivo*, our findings establish a novel paradigm of pathogen effector-mediated autophagy induction. The observed interactions align with the multistep processing of BraATG8 proteins during autophagosome formation. We propose that DM459 facilitates early autophagosome assembly by enhancing BraATG8’s engagement with BraATG4 (for precursor processing) and BraATG7 (for conjugation). This mechanism contrasts sharply with the autophagy-suppressive activity of *P. infestans* PexRD54, which disrupts ATG8 function in potato. This functional divergence may be attributed to the distinct biological contexts of these effectors: DM459 originates from *H. parasitica*, a pathogen adapted to *Brassica* crops, whereas PexRD54 is derived from *P. infestans*, which infects *solanaceous* plants. Currently, there is limited comparative genomic or functional analysis of DM459-like effectors across different races or isolates of *H. parasitica*. Thus, it remains unclear whether DM459 orthologs are widely conserved and whether they consistently activate autophagy in other pathosystems. We fully recognize this as an important area for future investigation and plan to conduct systematic studies to elucidate the precise mechanism by which DM459 modulates the autophagy pathway. Thus, DM459-induced host autophagy represents a unique example of an oomycete effector potentiating host resistance, likely reflecting an evolutionary adaptation in the ongoing plant–pathogen arms race.

### Alterations in BraATG8i-mediated autophagy contribute to the contrasting downy mildew resistance in *Brassica rapa*

In this study, we employed the resistant *B. rapa* line CC031^R^ to investigate autophagy-mediated defense responses against *H. parasitica*. Transient expression of *DM459* in CC031^R^ cotyledons induced distinct MDC accumulation patterns compared to empty vector controls, indicating altered autophagic activity. These findings establish a functional connection between autophagy modulation and plant immunity during *H. parasitica* infection. Consistent with the well-documented role of autophagy in plant defense, our results support the hypothesis that elevated autophagic flux in CC031^R^ underlies its enhanced resistance phenotype.

Our data identify SA as a potent inducer of *BraATG8i* expression (Fig. 7C). The resistant CC031^R^ line exhibited more rapid and pronounced *BraATG8i* induction compared to the susceptible CC032^S^ line, correlating with observed differences in autophagic activity. *H. parasitica* infection triggered significantly higher SA accumulation in the resistant line, a pattern replicated by *DM459* transient expression in both genotypes (Fig. 7A and B). These results suggest that DM459 may modulate SA biosynthesis, thereby enhancing both *BraATG8i* expression and autophagic activity.

Comparative sequence analysis revealed conserved coding regions but divergent promoter architectures between *BraATG8i^S^* and *BraATG8i^R^* alleles. An additional *as-1* cis-element was identified in the *BraATG8i^R^* promoter region, known as an SA-responsive element recognized by TGA transcription factors. In resistant lines, the promoter of *BraATG8i* contains an additional *as-1* cis-element, which confers stronger promoter activity and consequently higher BraATG8i accumulation. This results in an elevated level of autophagy compared to susceptible plants. When *BraATG8i* is overexpressed in susceptible lines, the increased availability of BraATG8i facilitates a stronger autophagic response upon recognition of DM459 during pathogen infection, thereby enhancing resistance. This finding aligns with established mechanisms of TGA-mediated autophagy regulation [28]. Through BiFC and Y1H assays, we identified three BraTGA transcription factors as direct regulators of *BraATG8i*, establishing a molecular link between SA signaling and autophagy activation.

### Conclusion

**Figure 8:**
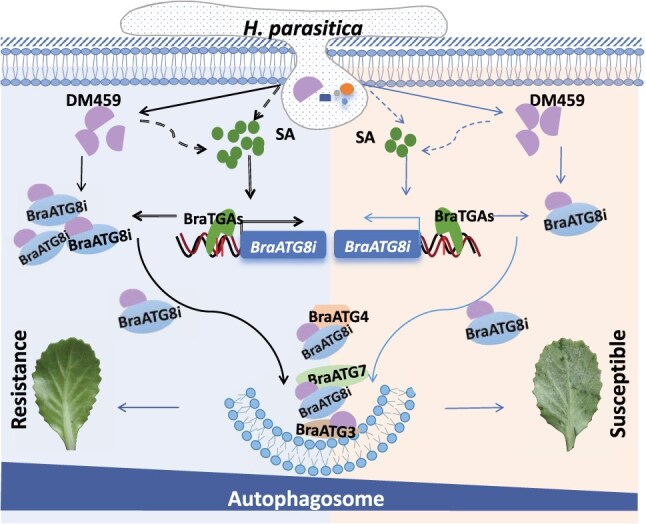
A working model for *BraATG8i*-mediated resistance enhancement in *B. rapa* against *H. parasitica*. Upon infection, the pathogen delivers effector proteins, including DM459, into the host cytoplasm via haustoria. In resistant lines, DM459 is recognized by BraATG8i, leading to the activation of autophagy. This process is potentiated by an SA-responsive feedback loop: SA signaling is amplified upon infection, and the presence of an *as-1* cis-element in the *BraATG8i* promoter enables its strong transcriptional upregulation by BraTGA transcription factors. The resulting high levels of BraATG8i protein facilitate more efficient recognition of DM459, further boosting autophagic activity and ultimately conferring strong disease resistance. In contrast, in susceptible lines, the expression of *BraATG8i* remains low due to a less responsive promoter, failing to initiate a sufficiently robust autophagic response, which results in compromised resistance.

Based on our findings, we propose a mechanistic model for DM459-triggered, BraATG8i-mediated resistance to *H. parasitica* (Fig. 8). Following infection, *H. parasitica* delivers effectors, including DM459, into host cells via haustoria. In the cytoplasm, DM459 interacts with BraATG8, enhancing its association with BraATG7 to promote autophagosome formation. In resistant lines, the *BraATG8i^R^* promoter’s *as-1* element enables robust SA-responsive expression, creating a positive feedback loop that amplifies both autophagy and effector recognition. This contrasts with susceptible lines, where limited *BraATG8i* expression results in attenuated autophagic responses. The nuclear localization of DM459 suggests additional regulatory functions, potentially influencing SA biosynthetic gene expression either directly or through interactions with host R proteins. Future studies should explore these nuclear mechanisms to fully elucidate DM459’s multifaceted contributions to plant immunity.

## Materials and methods

### Plant materials and growth conditions

The downy mildew-resistant *B. rapa* lines CC031^R^ (536 Qian) and susceptible lines CC032^S^ (Jiaoerye) were used in this study. *N. benthamiana* was employed for protein interaction assays. All *B. rapa* seeds were germinated and grown in pots containing a peat–vermiculite mixture (3:1, v/v) in greenhouse conditions. Plants were inoculated with *H. parasitica* at the one- to two-true-leaf stage. *N. benthamiana* plants were grown under similar conditions and used for *Agrobacterium tumefaciens* infiltration at the six-leaf stage. All plant materials were maintained in the greenhouse facility at the Vegetable Research Center, Beijing Academy of Agricultural and Forestry Sciences, under controlled conditions (22°C, 70% relative humidity, 16-hour light/8-hour dark photoperiod).

### Pathogen inoculation and disease assessment

*H. parasitica*, a biotrophic oomycete pathogen, was used for inoculation following established protocols [42]. Following the application of a fresh spore suspension (5 × 10^4^ zoospores/ml) to the abaxial leaf surface, inoculated plants were maintained in darkness with high humidity for 24 hours, incubated for 5 days under light, and then exposed to a further 12 hours of dark and humid conditions prior to disease evaluation at 7 dpi. Leaf samples were collected at 0, 12, 24, 72, and 120 hpi for subsequent analyses. Disease resistance was evaluated based on lesion development and sporulation intensity at 7 dpi. Pathogen content was assessed by qPCR quantification of the *H. parasitica* ITS region, normalized to the plant *GAPDH* gene, using total DNA extracted from surface-sterilized leaf discs.

### RNA isolation and quantitative reverse transcription PCR analysis

Expression analysis of *DM459* or *BraATG8i* in different samples from *B. rapa* was examined by qRT-PCR. Total RNA was isolated from 3-week-old plants grown in soil with a plant RNA extraction kit (Huayueyang, Polysaccharide Polyphenols Plant RNA Extraction Kit). RNA was used to synthesize the oligo(dT)-primed first-strand cDNA using the cDNA synthesis kit (TAKARA PrimeScript™ RT Reagent Kit with gDNA Eraser). Quantitative RT-PCR was performed with SYBR Green PCR Master Mix (Applied Biosystems, 4312704) on an Applied Biosystems 7500. The housekeeping gene *GAPDH* (*BraA06g009730.3C*) was used as an internal reference. The relative mRNA expression was calculated using the 2^−ΔΔCt^ method [43]. All the gene-specific primers used for qRT-PCR are listed in Table S1.

### Secretory function analysis

The 1- to 150-bp sequence of *DM459* containing signal peptide sequence (1–75 bp) was subcloned into the pSUC2 vector. pSUC2-AVR1b, pSUC2-Mg87, and YTK12 were used as positive, negative, and empty controls. The plasmid pSUC2-DM459, pSUC2-Mg87, or pSUC2-AVR1b was transformed into yeast strain YTK12. The resulting strains were subsequently grown on CMD-W medium for 5 days at 30°C. Secretory function was tested by growth assays on YPRAA medium. Yeast strains YTK12 carrying pSUC2-DM459, pSUC2-Mg87, or pSUC2-AVR1b were inoculated into liquid CMD-W medium, while the plasmid-free YTK12 strain was grown in YPDA liquid medium. All cultures were incubated at 30°C with shaking at 220 rpm for 24 hours. Cells were harvested, washed three times with sterile water, and resuspended. To each sample, 250 μl of 10 mM sodium acetate–acetic acid buffer (pH 4.7) and 500 μl of 10% (w/v) sucrose solution were added, followed by incubation in a 37°C water bath for 10 minutes. After centrifugation at 12000 rpm for 1 minute, 100 μl of the supernatant was transferred to a glass tube containing 900 μl of 0.1% TTC solution. The mixture was incubated at room temperature for 5 minutes, and color development was recorded. The PCR primers used for the plasmid construct of secretory function assays are given in Supplementary Table S1.

### Trypan blue staining

The cell death was detected using Trypan Blue Staining Kit (Coolaber, SL7121) according to the protocol handbook instructions. The cotyledons of *B. rapa* were collected and immersed in trypan blue staining solution. The samples were vacuum infiltrated (−0.08 MPa) for 1 hour and subsequently incubated in the staining solution at 37°C with gentle shaking for 12 hours. After staining, the samples were destained with 75% ethanol and imaged under a bright-field microscope.

### Subcellular localization analysis

The coding sequences of *DM459* and *BraATG8i* were subcloned into the pCambia2300-35S-GFP vector, respectively. pCambia2300-35S-H2B-mCherry and pCambia2300-35S-PIP2A-mCherry were used for the nucleus and plasma membrane (red) markers. All constructs were transformed into *Agrobacterium* strain GV3101. GV3101 carrying pCambia2300-35S-GFP (35S-GFP), pCambia2300-35S-DM459-GFP (35S-DM459-GFP), pCambia2300-35S-GFP-DM459 (35S-GFP-DM459), pCambia2300-35S-BraATG8i-GFP (35S-BraATG8i-GFP), pCambia2300-35S-H2B-mCherry, or pCambia2300-35S-PIP2A-mCherry constructs were cultured and resuspended in infiltration buffer (10 mM MgCl₂, 10 mM MES (2-(N-morpholino) ethanesulfonic acid), 150 μM acetosyringone) as previously described [44]. The bacterial suspensions (OD_600_ = 0.5) were infiltrated into 4-week-old *N. benthamiana* leaves. Fluorescence was examined 72 hours postinfiltration using an Olympus BX51 fluorescence microscope (Olympus Corporation, Tokyo, Japan) with excitation/emission settings of 488/507 nm for GFP and 588/610 nm for mCherry. The PCR primers used for the plasmid construct of subcellular localization analysis are given in Supplementary Table S1. The analysis was performed in triplicate.

### Protein–protein interaction assays

#### Yeast two-hybrid assay

Y2H assays were conducted as described previously [45]. The coding sequences of *DM459* were subcloned into bait vector pGBKT7. The coding sequences of *BraATG8i*, *BraATG4*, *BraATG7*, and *BraATG3* were subcloned into prey vector pGADT7, respectively. A pair of bait and prey plasmids was co-transformed into yeast strain AH109 using the PEG/LiAc method. The resulting strains were subsequently grown on SD/−Leu/−Trp medium for 5 days at 30°C. Potential protein–protein interactions were tested by growth assays on SD/−Leu/−Trp/−His/−Ade medium supplemented with X-α-Gal. Plasmid combinations pGBKT7–53/pGADT7-T and pGBKT7-Lam/pGADT7-T were the positive and negative controls, respectively. The PCR primers used for the plasmid construct of Y2H assays are given in Supplementary Table S1.

#### Bimolecular fluorescence complementation

The coding sequences of *DM459* were subcloned into vector pSPYCE. The full-length coding sequences of *BraATG8i*, *BraATG8a*, *BraATG8d*, and *BraATG8g* were subcloned into vector pSPYNE, respectively. All constructs were transformed into *Agrobacterium* strain GV3101. GV3101 carrying pSPYCE or pSPYNE constructs were cultured and resuspended in infiltration buffer (10 mM MgCl_2_, 10 mM MES, 150 μM acetosyringone) as previously described [44]. The designed pairs of constructs were infiltrated into 4-week-old tobacco leaf. Fluorescence was detected 72 hours postinfiltration using an Olympus BX51 fluorescence microscope (Olympus Corporation, Tokyo, Japan) with 488/507 nm. The PCR primers used for the plasmid construct of BiFC assays are given in Supplementary Table S1.

#### Luciferase complementation imaging

LCI assays were conducted as described previously [44]. The coding sequences of *DM459* were subcloned into vector pCAMIA1300-cLuc. The coding sequences of *BraATG8i*, *BraATG4*, *BraATG7*, and *BraATG3* were subcloned into vector pCAMIA1300-nLuc, respectively. All constructs were transformed into *Agrobacterium* strain GV3101. GV3101 carrying pCAMIA1300-cLuc or pCAMIA1300-nLuc constructs were cultured and resuspended as described in BiFC. The designed pairs of constructs were infiltrated into different positions of the same 4-week-old tobacco leaf. Three days after infiltration, the tobacco leaves were sprayed with luciferin solution (100 mM d-luciferin free acid, Promega), and Luc activity was captured in the dark for 7 min with a low-light cooled CCD imaging apparatus (Night SHADE LB985 with Indigo software). The PCR primers used for the plasmid construct of LCI assays are given in Supplementary Table S1. The protein interaction assays were performed in triplicate.

#### Co-immunoprecipitation

Co-IP assays were conducted as described previously [44]. The coding sequences of *DM459* were subcloned into vector pCAMIA1300-cmyc and pCAMIA1300-GFP, respectively. The coding sequences of BraATG8i were subcloned into vector pCAMIA1300-GFP. The coding sequences of BraATG4, BraATG7, and BraATG3 were subcloned into vector pCAMIA1300-flag, respectively. All constructs were transformed into *Agrobacterium* strain GV3101. GV3101 carrying pCAMIA1300-cmyc, pCAMIA1300-GFP, or pCAMIA1300-flag constructs was cultured and resuspended as described in BiFC. The designed pairs of constructs were infiltrated into 4-week-old tobacco leaf. Three days after infiltration, the tobacco leaves were harvested. The proteins were extracted using Plant Protein Extraction Kit (Cwbio, CW0885) according to the protocol handbook instructions. Immunoprecipitation **(**IP), 15 μl of anti-GFP affinity gel (MBL), was transferred to a sterile 1.5-ml microcentrifuge tube and washed three times with 1 ml of phosphate-buffered saline (PBS). For each wash, the suspension was gently inverted to mix, then centrifuged at 7000 rpm for 30 seconds, followed by careful removal of the supernatant. The equilibrated gel was then resuspended in 1 ml of prepared protein extract and incubated overnight at 4°C with continuous rotation. After incubation, the gel was collected by centrifugation at 7000 rpm for 30 seconds, and the supernatant was discarded. The gel was subsequently washed three times with 1 ml of PBS under the same centrifugation conditions. The final immunoprecipitated complex was resuspended in 50 μl of SDS-PAGE loading buffer and denatured by boiling for 5 minutes prior to western blot analysis. All proteins were detected by immunoblotting with anti-GFP/cmyc/flag antibodies (Abcam). The PCR primers used for the plasmid construct of Co-IP assays are given in Supplementary Table S1.

#### Yeast three-hybrid assay

The pBridge vector system was employed to analyze the interaction. The coding sequence of *BraATG8i* was subcloned into the pGADT7 vector. The coding sequences of *DM459*, *BraATG3*, and *BraATG7* were subcloned into the pBridge vector, respectively. Yeast strain AH109 was co-transformed with the following plasmid pairs: pGADT7-BraATG8i and either pBridge-BraATG3, pBridge-BraATG3-DM459, pBridge-BraATG7, or pBridge-BraATG7-DM459. The resulting strains were subsequently grown on SD/−Leu/−Trp or SD/−Leu/−Trp/−Met medium for 5 days at 30°C. Potential interactions were tested by growth assays on SD/−Leu/−Trp/−His/−Ade or SD/−Leu/−Trp/−Met/−His/−Ade medium. Plasmid combinations pGADT7 and all pBridge vectors were the negative controls. The PCR primers used for the plasmid construct of Y3H assays are given in Supplementary Table S1.

### Transient and stable expression

Full-length coding sequences of *BraATG8i* and *DM459* were subcloned into the pCambia2300-35S-GFP vector. All constructs were transformed into *Agrobacterium* strain GV3101. For transient expression, the bacterial suspensions (OD_600_ = 0.2–0.3) were infiltrated into 10-day-old *B. rapa* cotyledons. For stable transformation, the floral dip method was employed as previously described [44]. Transgenic plants were selected on appropriate antibiotic media. The PCR primers used for the plasmid construct of transient and stable expression assays are given in Supplementary Table S1.

### RNAi

A candidate fragment suitable for RNAi targeting was selected from the *BraATG8i* coding sequence using the VIGS-TOOL (https://solgenomics.net/vigs-tool). Specific primers (RNAi-F:TACAATTACCATGGGGTCATTCAAGGAAGAATTGACGT, RNAi-R:TCATCGATTGGGCGCGCTGTAGCACATGTAAACGAATCC) and primers (RCRNAi-F:GATCTCTTTGATGGGCTGTAGCACATGTAAACGAATCC, RCRNAi-R:GACTCTAGGGACTAGTCATTCAAGGAAGAATTACGT) were designed to amplify the selected fragment, which was subsequently cloned into the pFGC1008 vector for RNAi plant transformation. The procedure for generating transgenic plants was as described previously for stable overexpression.

### Autophagosome detection

All leaves used for autophagosome detection were harvested and treated with E64-d (50 μg/ml) for 30 minutes to stabilize autophagic structures in the dark. For MDC staining, leaf discs were incubated in 50 μM MDC solution for 30 minutes, washed with PBS, and observed under fluorescence microscopy. For TEM, samples were fixed in 2.5% glutaraldehyde, postfixed in 1% osmium tetroxide, dehydrated in an ethanol series, and embedded in Spurr’s resin. Ultrathin sections were examined using a Hitachi H-7650 TEM.

### Promoter activity analysis

The 2-kb promoter region of *BraATG8i* from CC031^R^ was subcloned into the *pro:GUS* vector. The constructs were transformed into *Agrobacterium* strain GV3101. GV3101 carrying *pro:GUS* or *proBraATG8i:GUS* constructs were cultured and resuspended as described in BiFC. The bacterial suspensions (OD_600_ = 0.5) were infiltrated into 4-week-old tobacco leaves. Promoter activity was detected using GUS Staining Kit (Coolaber, SL7160) according to the protocol handbook instructions. Three days after infiltration, leaf discs (0.5 cm in diameter) were collected from the infiltrated areas of tobacco or *B. rapa* leaves using a biopsy punch and immersed in GUS staining solution containing X-Gluc substrate. The samples were vacuum infiltrated (−0.08 MPa) for 1 hour and subsequently incubated in the staining solution at 37°C with gentle shaking for 12 hours. After staining, the leaf discs were destained with 75% ethanol and imaged under a bright-field microscope. The PCR primers used for the plasmid construct of promoter activity assays are given in Supplementary Table S1.

### Protein–DNA interaction assays

#### Luciferase reporter assay

The 2-kb promoter regions of *BraATG8i* from CC031^R^ and CC032^S^ were subcloned into the *pro:Luc* vector. The CDS sequences of *BraATG* (*BraA03g035190*, *BraA08g026970*, *BraA07g026840*) were subcloned into the pCambia2300-35S-GFP vector. All constructs were transformed into *Agrobacterium* strain GV3101. *Agrobacterium* carrying *proBraATG8i^R^:GUS* or *proBraATG8i^S^:GUS* constructs was cultured and resuspended as described in BiFC. The designed pairs of constructs were infiltrated into different positions of the same 4-week-old tobacco leaf. Three days after infiltration, the tobacco leaves were sprayed with luciferin solution (100 mM d-luciferin free acid, Promega), and Luc activity was captured in the dark for 7 min with a low-light cooled CCD imaging apparatus (Night SHADE LB985 with Indigo software). The PCR primers used for the plasmid construct of Luc assays are given in Supplementary Table S1. The protein interaction assays were performed in triplicate.

#### Yeast one-hybrid assay

The 2-kb promoter region of *BraATG8i* was subcloned into the pHis vector. The CDS sequences of *BraATG* (*BraA03g035190*, *BraA08g026970*, *BraA07g026840*) were subcloned into the pGADT7 vector. The pGADT7-TGA1/2/3 and pGADT7 empty vector then co-transformed with the recombinant *pHis:BraATG8i* vector into the Y187 yeast strain. The resulting strains were subsequently grown on SD/−Leu/−Trp medium for 5 days at 30°C. Potential protein–DNA interactions were tested by growth assays on SD/−Leu/−Trp/−His medium supplemented with 150 mM 3-AT. Plasmid combination *pHis:BraATG8i*/pGADT7 was the negative control. The PCR primers used for the plasmid construct of Y1H assays are given in Supplementary Table S1.

## Supplementary Material

Web_Material_uhaf358

## Data Availability

All data required for evaluating the conclusions in the paper are available. All materials generated in this study are available from the corresponding author S.C.Y.
