# Supplementary material for: Targeting and activation of BraATG8i by an RXLR effector DM459 contribute to downy mildew resistance in Brassica rapa
Source: Hortic Res. 2025 Dec 31;13(4):uhaf358. doi: 10.1093/hr/uhaf358 (PMC13091401; doi:10.1093/hr/uhaf358)
Supplement: Web_Material_uhaf358 [file web_material_uhaf358.zip › Supplementary Figure.pdf]

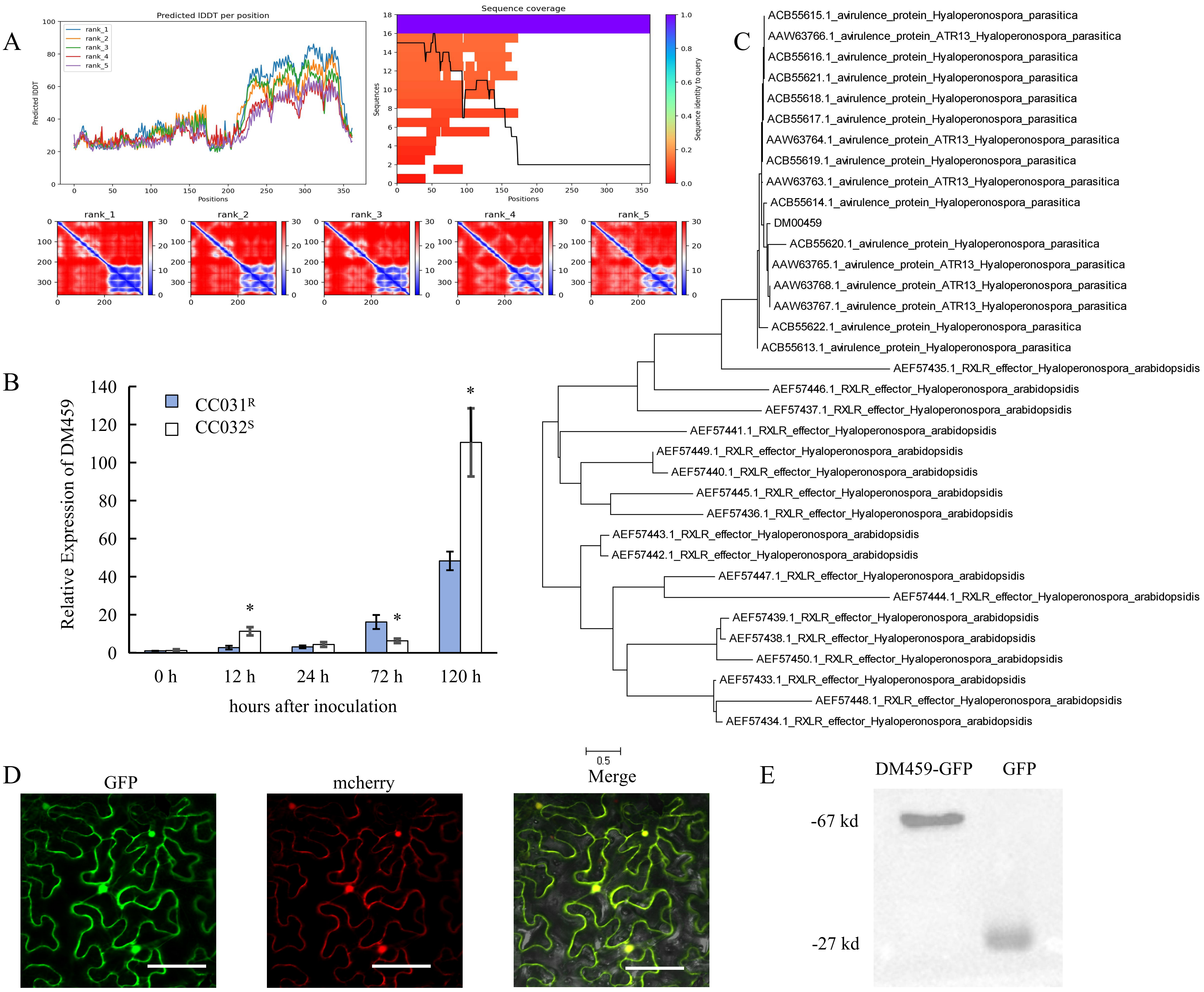

Fig. S1 DM459 was a RxLR effector. (A) Predicted three-dimensional structure of DM459 generated by AlphaFold2. (B) Temporal expression pattern of DM459 in *B. rapa* after inoculation with *H. parasitica*. (c) Phylogenetic analysis of DM459. A maximum-likelihood phylogeny was reconstructed to illustrate the relationship of DM459 to canonical RxLR effectors. (D) Subcellular localization of GFP in *N. benthamiana*. GFP were transiently expressed via agrobacterium infiltration. Images were taken 48-72 hours post-infiltration. Bars, 100 µm. (E) Immunoblot analysis confirming the integrity of GFP-tagged proteins. Total proteins were extracted from *N. benthamiana* leaves expressing GFP or DM459-GFP. The blot was probed with an anti-GFP antibody, demonstrating full-length fusion protein without degradation.

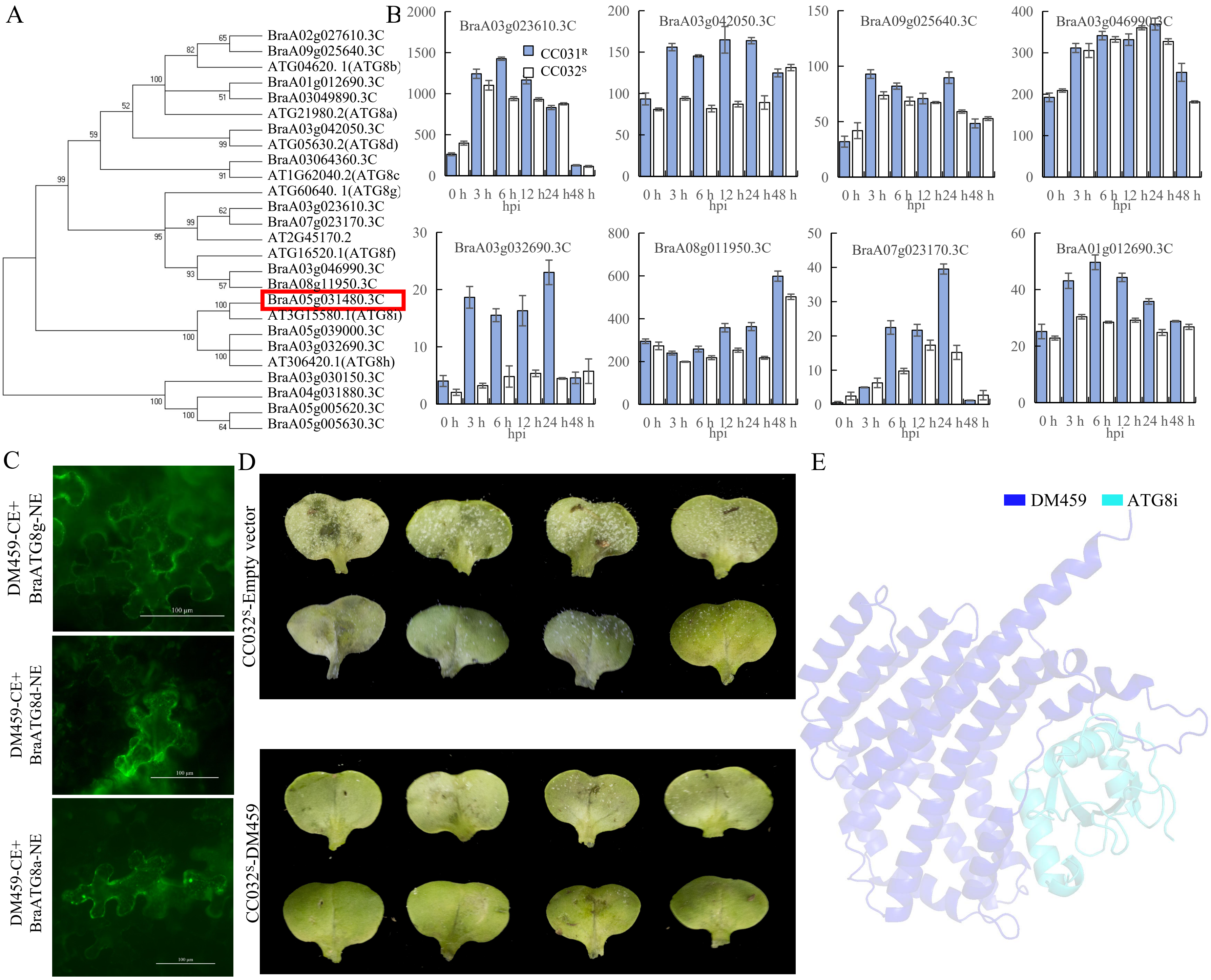

Fig. S2 The effector DM459 interacted with BraATG8s. (A) Phylogenetic analysis of BraATG8i. A sequence alignment of ATG8 family genes from *B. rapa* and *A. thaliana* identified BraA05g031480.3C as the ortholog of AtATG8i, hereafter named BraATG8i. (B) Transcriptional induction of *BraATG8* genes by *H. parasitica*. Transcriptome profiling of the susceptible (CC032^{S}) and resistant (CC031^{R}) *B. rapa* lines at various time points post-inoculation revealed upregulation of multiple BraATG8 family members, suggesting their involvement in the response to downy mildew. (C) DM459 interacts with multiple BraATG8 proteins in planta. The interaction between DM459 and three selected BraATG8 family members (BraATG8a, f, i) was confirmed by bimolecular fluorescence complementation (BiFC) assay in *N. benthamiana* leaves. Reconstituted YFP fluorescence indicates protein-protein interaction. Bars, 100 µm. (D) Transient expression of DM459 enhances disease resistance. *B. rapa* cotyledons transiently expressing DM459-GFP exhibited enhanced resistance to *H. parasitica* compared to the GFP-only control, as assessed by symptom development at 7 dpi. (E) Structural prediction of the DM459-braatg8i interaction by AlphaFold3.

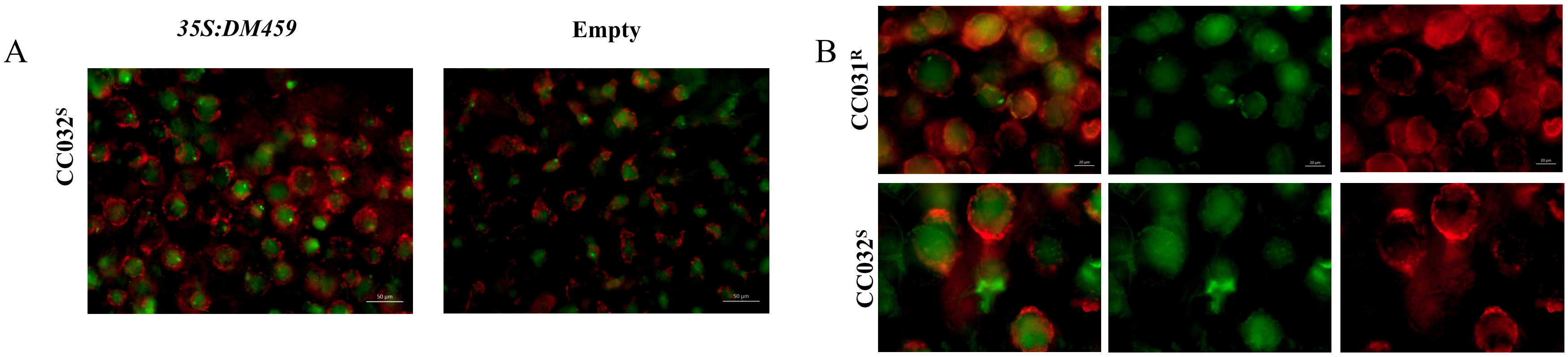

Fig. S3 ***H. parasitica*** **infection and the effector DM459 induce autophagy in** ***B.rapa*****.** (A) Transient expression of *DM459* enhances autophagic activity. The susceptible line CC032^S was transiently transformed with a DM459-GFP construct or a GFP control. Autophagic structures were visualized by monodansylcadaverine (MDC) staining and observed via confocal microscopy 3 days post-infiltration. Bar, 50 µm. (B) *H. parasitica* infection triggers host autophagy. Autophagic activity in the susceptible line CC032^S was monitored by MDC staining at various time points after inoculation with *H. parasitica*. Images show a representative sample from an infected leaf. Bar, 50 µm.

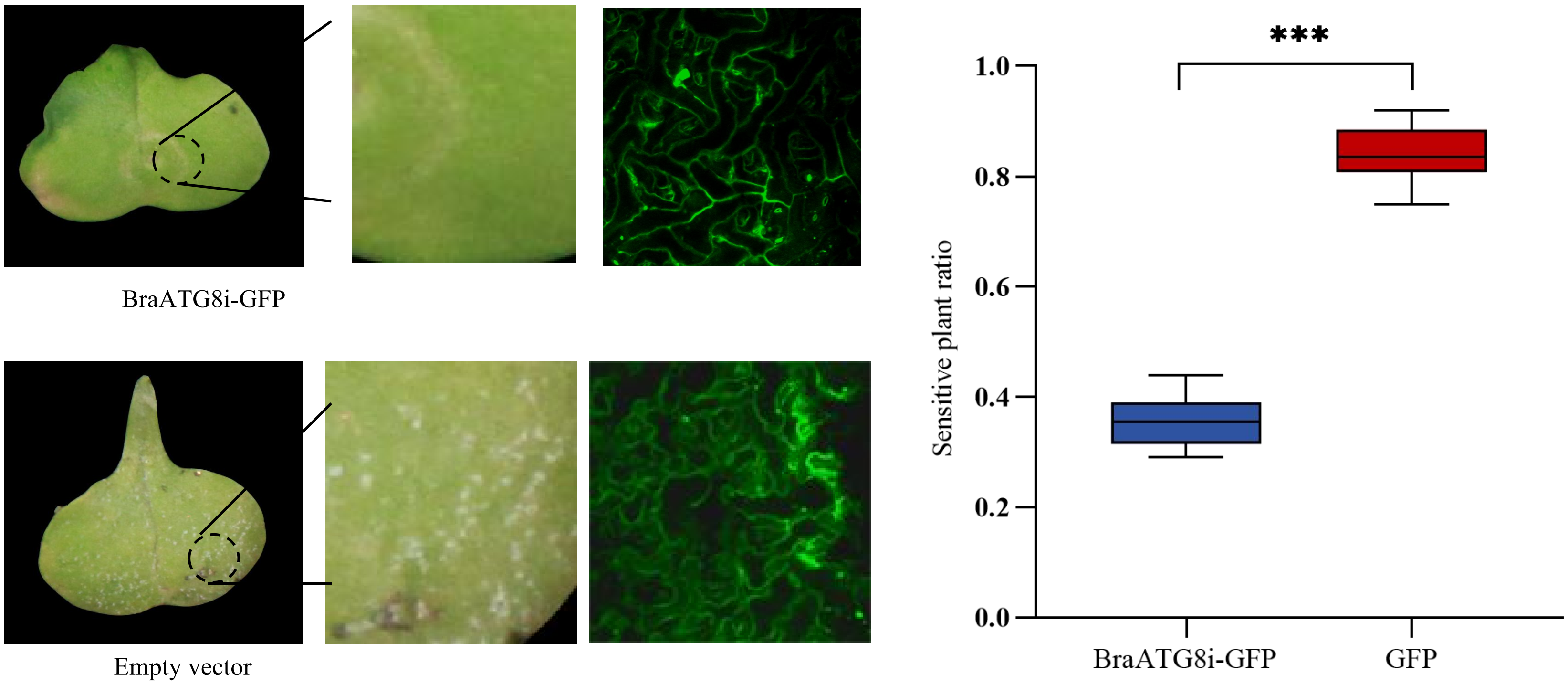

Fig. S4 Transient expression of *BraATG8i* enhance resistance to downy mildew in *B. rapa*. Disease phenotype of BraATG8i-expressing cotyledons. *B. rapa* cotyledons transiently expressing BraATG8i-GFP or a GFP control were inoculated with *H. parasitica*. Photographs were taken 7 dpi to assess disease symptoms. The incidence of *H. parasitica* infection was recorded and calculated from 50 cotyledons per treatment (as shown in panel a). Data are presented as mean ±SD. Statistical significance was determined by a two-tailed Student's t-test (***$P < 0.001$).

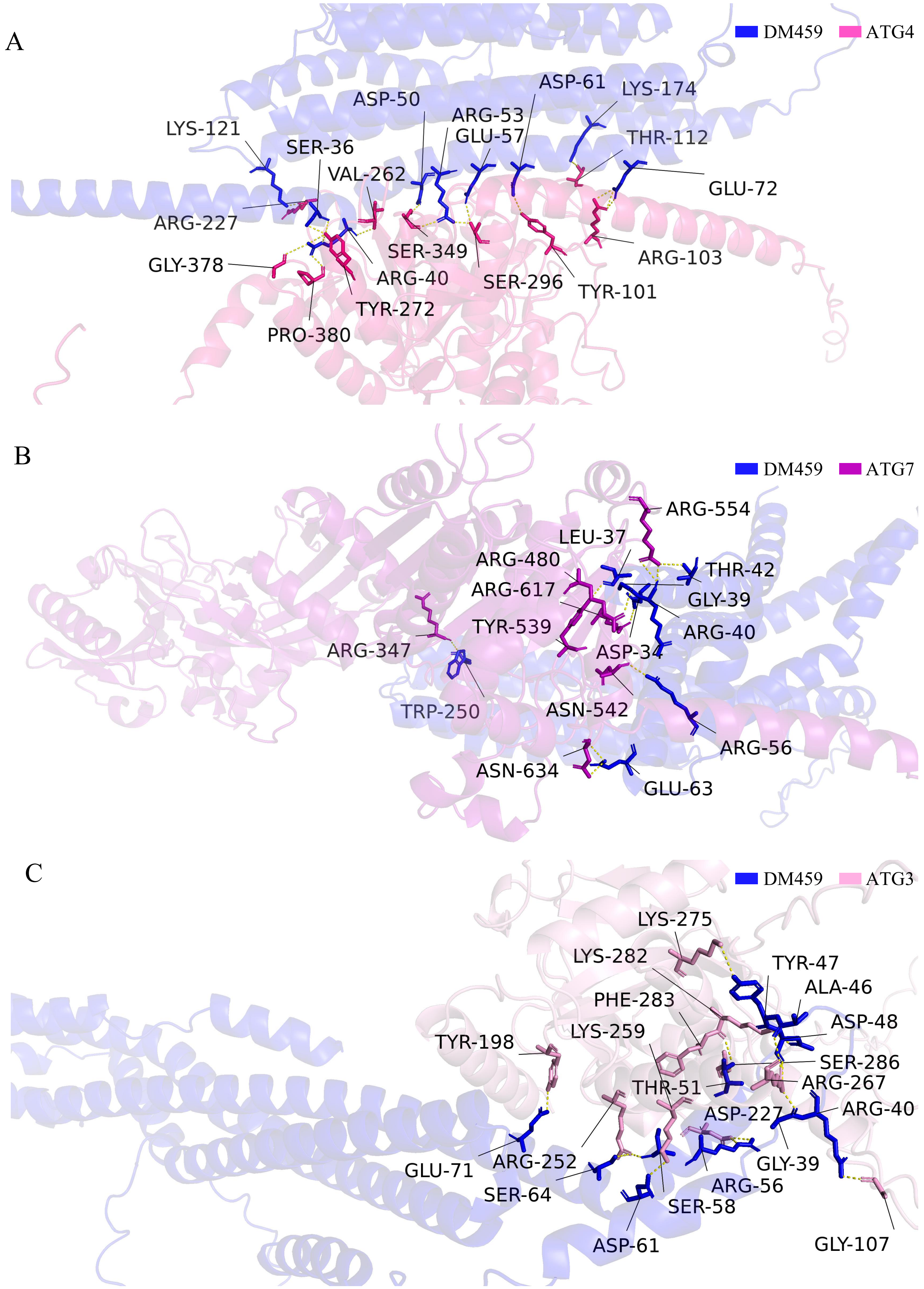

Fig. S5 The interaction between DM459 and other BraATGs. (A) The interaction between DM459 and BraATG4. (B) The interaction between DM459 and BraATG7. (C) The interaction between DM459 and BraATG3.

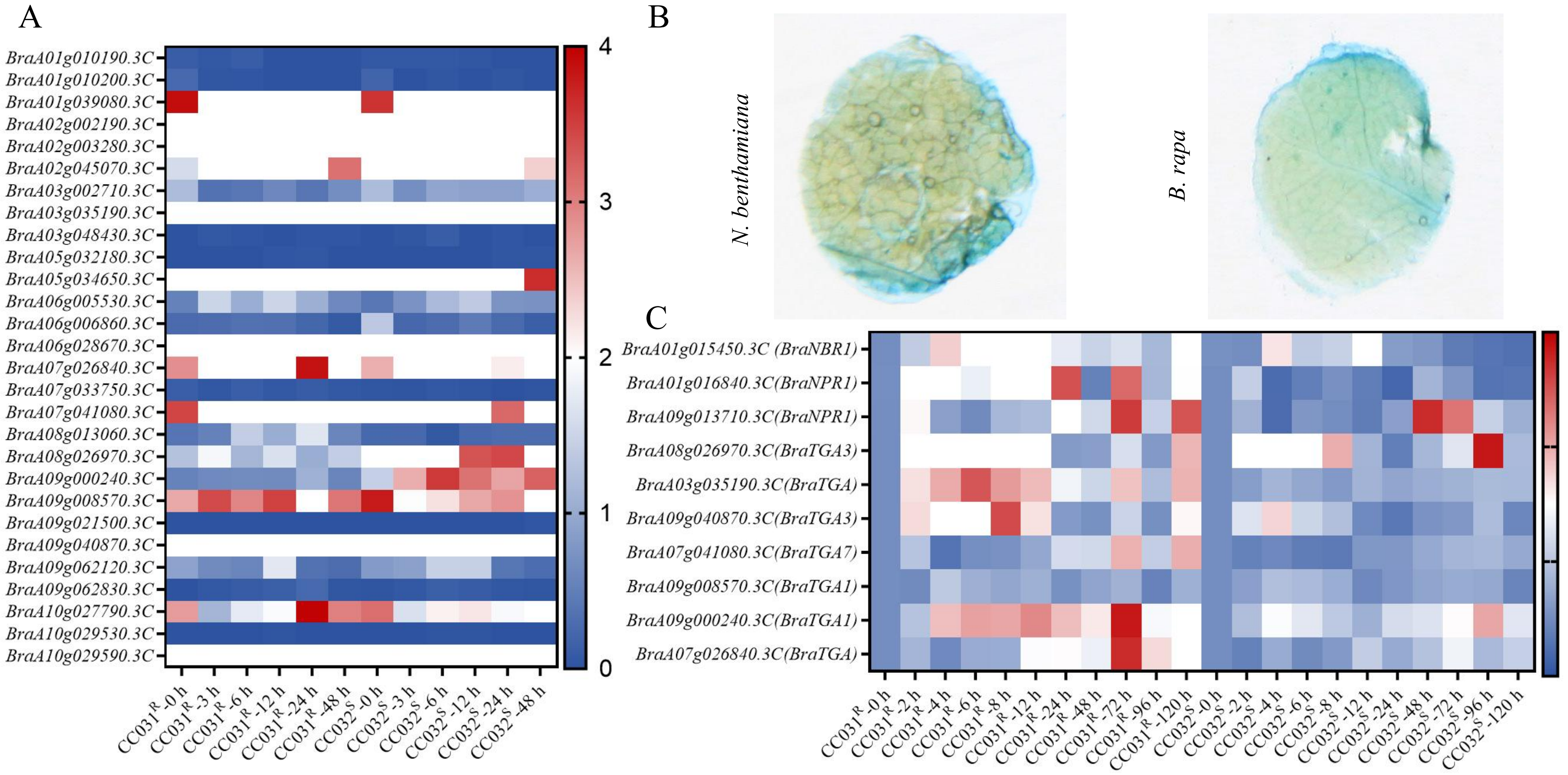

Fig. S6 SA signaling pathway is hyperactivated in resistant line. (A) Expression dynamics of SA biosynthesis and signaling components after pathogen challenge. Transcript levels of key SA biosynthesis genes and BraTGA transcription factors were analyzed by RNA-seq or qRT-PCR in the susceptible (CC032^{S}) and resistant (CC031^{R}) lines at indicated hours post-inoculation with *H. parasitica*. (B) Activity of the BraATG8i promoter in different systems. Promoter activity was assessed by measuring the expression of GUS driven by the BraATG8i promoter in transiently transformed *N. benthamiana* leaves and *B. rapa* lines, n=20. (C) SA treatment induces the expression of key autophagy and signaling components. Transcript levels of *BraNBR1*, *BraNPR1*, and *BraTGA* genes were quantified by qRT-PCR in CC032^{S} and CC031^{R} lines at various time points after foliar application of 20 mM SA. *GAPDH* was used as an internal control. Data are presented as mean $\pm$SD (n=5 biological replicates).
